# The medicinal recipes of Hannah Woolley: everyday practice and female authority in seventeenth-century England

**DOI:** 10.1590/S0104-59702023000100007en

**Published:** 2023-04-03

**Authors:** Marina Juliana de Oliveira Soares

**Affiliations:** i Professor of Education in the Humanities, Faculdade SESI-São Paulo. São Paulo – SP – Brazil oliveiras.mari@gmail.com

**Keywords:** Hannah Woolley (c.1623-c.1677), seventeenth century, recipe book, medical practice, women healers

## Abstract

This article describes a seventeenth-century English woman writer’s interests in medical care and the reasons that led her to publish texts on this topic. Hannah Woolley offered guidance on a wide variety of topics in the domestic sphere, including recipes for health and beauty. Here we investigate the principles that governed the preparation of these recipes, Woolley’s intentions in writing on this topic, and the way in which academic medicine was translated and practiced by women routinely during this period. Defining these issues will help shed light on the scenario in which literate female healers worked and the nature of their relationships with learned physicians.

How were the medical theories that were popular in Western Europe during the modern period incorporated and practiced in everyday British life? And what tools were available and used to combat disease by normal women without medical training? These questions will guide us in the pages to follow; to answer them, I will utilize a text written and published by a seventeenth-century English woman that combined 111 medicinal recipes in a section dedicated to medicine and surgery. Although the publications of Hannah Woolley (c.1623-c.1677) are highlighted, it is important to recall that many medicinal recipes were produced by women during this period, as we shall see.

In order to assess Woolley’s medical recommendations, we must briefly outline the principles that guided medical activities in England at that time. For the purposes of this text, we are most interested in the healing procedures that were widely used by erudite and empirical physicians, and the space occupied by women within this scenario, whether learned or non-specialized. Likewise, it is necessary to investigate the importance of manuals containing medicinal recipes, and women’s participation in the authorship of these works.

Because these recipe books especially targeted high-class women, we must question how far their popularity extended among the female public, as well as the potential impact of this content on credentialed physicians. After all, as practitioners trained in universities, these men may have viewed the potential empirical activities encouraged by such works with suspicion, an attitude that gained traction in medical institutions such as the College of Physicians in London.

## Fundamentals of medical practice in modern Europe

When considering the era that marks the start of modern Europe, i.e., between 1500 and 1800 (Burke, 2009, p.xiv), we must recall the intense commercial and maritime movement that took place during this period. Especially from 1400 to 1600, Europeans were active in “oceanic exploration, the opening up of new trade routes and the development of new trades” (Arnold, 2002, p.61). Besides the violent repercussions of the colonization process, another movement was underway: interchanges related to nature, medicines, and curing practices between Europeans and native peoples. Previously unknown diseases drove Europeans to explore foreign herbs and drugs, broadening their repertoire in terms of the *materia medica* (Bleichmar cited in Schiebinger, Swan, 2005, p.83). Alongside the medicinal herbs incorporated into the pharmacopeia, the Europeans adopted practices like “moxibustion, tobacco smoking and chocolate drinking” (Lindemann, 2010, p.277). Harold Cook (1986, p.28) coined the term “medical marketplace” for this phenomenon, considering the variety of sources where medicines could be purchased in the seventeenth century.

Despite the constant changes in the external scenario and new theoretical formulations in the academic environment – such as the anatomical findings of Andreas Vesalius (1514-1564) and the discovery of blood circulation by William Harvey (1578-1657) –, the foundations of medicine that were in vogue in Europe at that time did not change (Conrad et al., 1995, p.415). Certainly, the medical curriculum was altered at this time, for example with innovations caused by “chemical and mechanical concepts” (Lindemann, 2010, p.141), but the principles that governed the teaching and practice of medicine continued to be “the body’s four humors^1^ and the attempt to maintain their balance” (Byrne, 2013, p.18).

Although the four bodily humors (phlegm, yellow bile, black bile, and blood) were cited in medical texts since at least Ancient Greece, the way these humors affected health or illness was systematically contemplated by the Greek physician Galen (Claudius Galenus, 129-c.216). Galen examined the Hippocratic texts in detail in his efforts to create a solid medical system based on the philosophical tradition, particularly Platonic ideals. Around 650, the medical theory and practice dominant in the Greek East and later, Islamic regions and the Latin West became known as Galenism (Nutton, 2013, p.299).

The “theory of the humors” can be summarized from three of its main supports: dietetics, pharmacology, and surgery (Jouanna, 2012, p.17). It is important to remember that to Galen, diet referred not only to food and drink but also aspects of an individual’s lifestyle including sleep, exercise, and surroundings (Nutton, 2013, p.246). The order of the therapeutic procedures above was established by Galen, namely considering dietetics as the first therapeutic approach, while surgery was understood to be the last resource utilized because of its invasive nature and the painful experience it produced (p.246). While Woolley used the term surgery in her work, her recommendations broadly referenced dietetics and pharmacology.

Food played a crucial role in humoralist medical theory, since after ingestion it “was turned into blood by the liver and distributed to the body by the veins” (Nutton, 2017, p.15). For this reason it was essential to understand the properties of foods (such as whether they were wet, dry, cold, or hot) in order to balance the humors and restore health. Although each patient’s temperament also needed to be determined to better guide therapy, one general recommendation in humoral theory was widespread: curing by opposites. This treatment involved ingesting foods or drinks with the opposite qualities of the patient’s illness. For example, a “morbidly chilled” stomach was “cured by a hot drug etc.” (Donini cited in Hankinson, 2008, p.61).

In broad terms, Galen attempted to classify drugs as simple or compound, indicating the differences between theoretical pharmacology and its practical applications. In this way, so-called “simple drugs” included herbs, animal products, or minerals that generated a “unique action” resulting from their connection to the cosmos. For example, “substances linked with the first of the Five Ages of Humankind” (according to mythology) heated the body, thus eliminating excess water (Glick, Livesey, Wallis, 2005, p.395). Since simple drugs demonstrated limited effectiveness against disease, it was necessary to use compound drugs which involved a mixture of ingredients. They were recommended when contrary forces needed to be applied simultaneously, such as in wounds caused by venomous creatures (Prioreschi, 1998, p.440).

## Medical recipe books

At the time when Hannah Woolley was writing her books on domestic subjects with medicinal recipes, English society already had a solid tradition of this literary genre. These books originated from “medieval books of secrets” that recreated “treasured medical, alchemical, or trade recipes” written in Latin that circulated among the elites (Field cited in Down, Eckerle, 2007, p.50). While men were responsible for the increasing publication of these books, women made their recipes known “exclusively in manuscript until the middle of the seventeenth century” (Field cited in Down, Eckerle, 2007, p.50). But printing women’s texts became viable and even lucrative after the Restoration in 1660; in this sense, it is important to remember that Woolley is considered the first woman to produce these publications in a “commercial enterprise” (Marchitello, Tribble, 2017, p.211), a fact that is closely connected to the popularity of this genre during the second half of the seventeenth century.

Lettered “middle class and elite” women were directed by their mothers, family members, and governesses to learn the “domestic arts,” which included culinary as well as medical subjects. In her autobiography, Lady Grace Mildway (c.1552-1620) recalled that her governess encouraged her to read William Turner’s *Herbal*, as well as a text on surgery. As an adult, Mildway compiled “many hundreds of medicinal recipes,” and was a healer to family members, friends, “and the poor in rural Northamptonshire” (Field cited in Down, Eckerle, 2007, p.52). The home consequently was a main site for medical care and simultaneously the center of female authority on issues of this nature (Stine, 1996, p.108).

The era in which Woolley wrote was preceded by a scenario that favored women’s writing. During the 1650 a new generation of female recipe authors emerged, including aristocratic women (Leong, 2018, p.150). Based on “secrets” from noble men and women, or offering recipes approved by erudite physicians, these works became popular among England’s literate classes and paved the way for Woolley’s frequent publications. The hybrid nature of her work (with culinary as well as medicinal recipes) led to specialization in the following years, a process that became more firmly established in the eighteenth century (Marchitello, Tribble, 2017, p.211).

The popularity of these books resulted from growing literacy in the country as well as decreases in the cost of paper and other raw materials. The texts circulated in print but also continued to be copied by hand. This genre was especially prolific during the seventeenth century. Between 1600 and 1700, book publishers in London “issued over 200 medical recipe titles,” with roughly sixty new texts and around 170 reprints, “making them one of the most popular genres of vernacular medical texts in early modern England” (Leong, 2018, p.13).

While it is impossible to precisely state the number of recipe books published during the seventeenth century throughout all of England, we can state with certainty that women were customary authors of this genre. The historian Jennifer Stine has estimated that approximately one hundred books were produced by women in early modern England. Stine (cited in Down, Eckerle, 2007, p.50) also suggests that many other anonymous recipe books in archives were written by women. One notable writer in this genre was Woolley, who was considered “the most popular domestic writer” of the late seventeenth century and “the first woman to publish a cookbook” (Wall, 2010, p.394).

We do not have specific details about Woolley’s biography, but we do know that she was orphaned at 14 years of age. Her musical abilities and knowledge of Italian subsequently captured the attention of a noblewoman who hired her as a governess. In this position, Woolley nurtured and developed her interests in subjects including medicine.

At around 24 years old, she married Benjamin Woolley, a teacher at a school in Essex, with whom she had four children. Despite domestic tasks and responsibility for roughly seventy students, she did not restrict herself to these duties: she began to dedicate herself to writing and publishing books on cooking. Her first cookbook, *The ladies’ directory*, was published in 1661; it was followed three years later by *The cook’s guide*, which also targeted female readers (Hartley, 2003, p.950). After the death of her first husband, Woolley saw publishing recipe books as a way to earn money and support her family (Shanahan, 2015, p.87); the result can be seen in the texts she published in the 1660s and 1670s.

After remarrying in 1666, Woolley did not abandon her writing career; in fact, her intellectual production intensified. One of her best-known works on household administration dates from this period. *The queen-like closet* was first published in 1670, reprinted four times, and translated into German in 1674 (Hartley, 2003, p.465). Although she indicated that the book was the result of knowledge acquired first-hand, Woolley still emphasized its scientific underpinnings. In a recipe for jelly for “a weak stomach,” she cited a conversation with a doctor in which she asked about musk and grey amber (Woolley, 1675b, p.110). Here and in other parts of the book, such references provide support for her recommendations.

Another popular book written during this period and attributed to Woolley^2^ is *The gentlewoman’s companion*, which was intended as a “guide for women.” The start of the text noted it was rare for a woman to be a writer, which was a “bold undertaking” (Woolley, 1673, p.10). This 1673 book included chapters dedicated to remedies for all types of illnesses or female inconveniences. Whether the issue was nosebleeds, stomachaches, or problems producing breastmilk, Woolley had suggestions to reduce the suffering of her readers or of any sick people they might happen to visit. After all, it was impossible to deal with sick people “without some knowledge in Physick, and the several operations of Herbs and Spices” (p.161).

Her concern with female education extrapolated to medical intent. To Woolley, women’s education was neglected across the board, as she wrote in *The gentlewoman’s companion* (Woolley, 1673, p.1). Even though she did not wish to stir a female rebellion, she defended the cultivation of knowledge so that women could educate their own children; we recall that she herself was supported by a noble lady after the death of her parents. Likewise, a mother or governess should be well-acquainted with the nature and disposition of children to better educate them (p.5). The female nature of her texts also referred to imbalances in relations between the sexes. She stated that there was no difference in the origin of women’s and men’s souls, and consequently women were just as capable of progress as men, through “good Education” (p.1).

Amid the success of her books, Woolley became a widow yet again, and in 1674 went to live in the home of Richard Woolley, probably her son. During this period she sold remedies at “reasonable Rates” and declared herself to training noble ladies who wished to undertake such service (Hartley, 2003, p.950). As we shall verify in the book that is the focus of this article, her intention was to provide women with the skills needed to prepare and potentially sell the remedies mentioned in the text. To do so, the exact quantity of ingredients as well as the method of preparation were detailed in each recipe. Besides the content, Woolley’s work was also considered a reference for its format. Most of her books offered a table of contents, which was adopted by cookbook writers in the eighteenth century like Elizabeth Raffald, Eliza Smith, and Martha Bradley (Wall cited in Smith, Wilson, 2011, p.171).

Her 1675 book entitled *The accomplish’d lady’s delight, in preserving physick, beautifying, and cookery* was divided into three sections and contained recipes for food as well as medicinal preparations and domestic guidance (Woolley, 1675a).^3^ The first section was dedicated to the preparation of syrups and jellies from fruit and flowers. The third section was a similarly organized guide to food preparation. The second part was particularly focused on health, with over forty pages of medicinal recipes. Although this medical specialization is most evident in part two, healing is mentioned in other places in the book, and consequently may help us understand the author’s intentions.

## Ancient and modern principles in medicinal recipes

The second part of the book, entitled “The physical cabinet,” begins with the following description: “Excellent Receipts in Physick and Chirurgery,” as well as “some Beautifying Waters, to Adorn and add Loveliness to the Face and Body.” Here I shall investigate the medicinal recipes and advice directed toward women in order to assess the role they played in everyday healing and what these female functions can tell us about the medicine which was popular in England during the second half of the seventeenth century.

Close reading of the medicinal recipes suggests not only that routine ingredients in cooking were used, but that common treatments in Hippocratic and Galenic theory were recommended. The humors are first mentioned in a recipe for vinegar syrup. Although it is found within the cooking recipe section, Woolley was clear about its use; it was recommended for bodies saturated with phlegm or resistant humors, since it worked to unblock the stomach, liver, spleen, and kidneys, making it possible to expel phlegm and yellow bile (Woolley, 1675a, p.82). In the text, yellow bile is referred to as “choler,” a reference to the ancient Greek *choli* that revels the link between this humor and the choleric temperament (Fountoulakis, 2015, p.163). Notably, vinegar is one of the most frequently mentioned ingredients in the text; one recommended use was against the plague, a disease that ravaged England during the seventeenth century.

The humors were mentioned again in the second part of the book in recipe number 28, against “Kings Evil.” The main ingredient in this formulation was “Broomflowers Distilled:” the patient was to ingest this liquid in the morning on an empty stomach, since it would help “purge the evil Humour” and help to internally shrink scrofula (Woolley, 1675a, p.137). The illness called Kings Evil referred to the swollen lymph glands known as scrofula, one manifestation of tuberculosis; its microbial cause would be determined in the nineteenth century by the German physician Robert Koch (Bertolli Filho, 2001, p.38). Since medieval times, the kings of France and England laid hands on scrofula with the intent of curing their subjects. But it was in modern times that the royal touch “assumed its determined place among the minutely regulated pomp the absolute monarchies surrounded themselves with” (Bloch, 1993, p.91).^4^

Additional evidence of antique medical authority was a recipe with a name believed to refer to the Greek physician Hippocrates (c.460-377 a.C.): hippocras. This preparation consisted of spiced wine, and was well-known since the late Middle Ages. *Vinum Hippocraticum*, as it came to be known among physicians and pharmacists, was considered a pleasant way “to improve one’s health” (Goldstein, 2015, p.333). Woolley’s book contained two recipes for this concoction. Since the author placed both recipes in part one, among various culinary preparations, there are no medical recommendations for its use. But both recipes produce a liquor (as the author describes it) from mixing wine with similar ingredients: sugar, cinnamon, ginger, and cloves (Woolley, 1675a, p.6-7, 37-38). Hippocras could be taken after meals, due to its alleged digestive functions, or even prescribed as an aphrodisiac, which was the case among several eighteenth-century doctors (Jianu, Barbu, 2018, p.362).

Liquid preparations are common throughout the book. Besides the constant use of wine, another common formulation in the medicine of this period was the cordial, distilled drinks that could be diluted with water before serving. These liquors likely originated in thirteenth-century Italy, and reached England in the fifteenth century, where they were known as “distilled cordials waters” (Bamforth, Ward, 2014, p.306). The medical benefits of cordials were not restricted to cardiac health (as suggested by the Latin name *cordis*); their medicinal properties were prescribed to treat numerous diseases. Certainly for this reason, no recommendations for use were provided alongside the recipe for the “most excellent cordial” recommended by Woolley, the “Catholicon” (universal, in Greek), since it was essentially a panacea.

Another variant of the cordial was the julep; although there is a single mention of this syrup (in recipe number 54), cordials are cited a dozen times in the book. The julep is derived from the Arabic word *julāb*, and in turn from the Persian *gul* (rose) and *āb* (water), and initially contained three ingredients: water, sugar, and distilled rose petal extract (Bouras-Vallianatos, 2020, p.165). Roses had been used as a medicine since Antiquity, and rosewater was widely traded by the Arabs and introduced to Europe around the second half of the tenth century (Touw, 1982, p.72). Considering how often roses were mentioned in the book (at least twenty times in parts one and two) and the increase in domestic cultivation of flowers in England since the sixteenth century (Thomas, 1984, p.223), they can be considered a routine ingredient in popular medicine.

The commercial and colonializing expansion at the start of the Modern Era “gave rise to a new world of interaction between Europeans, Asians, Africans and Amerindians” with subsequent exchanges of new drugs and ingredients in the “markets of Asia and Europe” (Chakrabarti, 2014, p.2). Spices were some of these foreign ingredients that were increasingly consumed in Europe as their prices fell. Portugal played an especially important role in this process, transporting seeds and plants between the East and West (Algranti, 2012, p.13).^5^ This directly impacted medical formulations, which began to add “multiple spices at once” (Azevedo, 2017, p.101). According to the theory of humors, spices were considered hot and dry, and consequently were to be used against cold and wet diseases (p.101). One of the recipes that incorporated spices was “Dr. Stevens Water,” a very popular preparation in the seventeenth century, which contained cinnamon, galanga, clove, and grains of paradise. Although the author mentions this recipe in part one, without any potential indications for use (Woolley, 1675a, p.12), it is important to recall the healing nature of this preparation. “Dr. Stevens Water” was found in recipes published before and after Woolley’ book. A 1667 book by an anonymous writer entitled *The ladies cabinet enlarged and opened* explained that “the principle use of this water” was against all illnesses related to the cold. It went on to state that Doctor Steven himself, who had been confined to his bed for ten days, healed himself with this compound and went on to live to age 98 (Azevedo, 2017, p.103).

A similar prescription, “Dr. Stephen water,” was another panacea “often recorded in recipe collections” (Stobart, 2016, p.40). Spices were also part of this formulation, which was recommended to comfort “vital spirits” and to combat “internal diseases” caused by the cold. The author also stated that this drink helped barren women to conceive, fended off parasites, and cured coughs, toothaches, and stomachaches (Woolley, 1675a, p.160).

Finally, there was Doctor Willoughby’s “aqua mirabilis” (Woolley, 1675a, p.161), probably a reference to the late-seventeenth-century Irish physician of that name.^6^ This “miraculous water” was a panacea made from spices blended in wine that dates back to the thirteenth century (Allen, 2016, p.101). A recipe for “aqua mirabilis” was already introduced in part one of the book (Woolley, 1675a, p.12) and included ingredients common in the recipes mentioned previously such as clove, galanga, cardamom, nutmeg, and ginger.

Imported ingredients appeared in two other formulations described in the book. One was “the Countess of Kents Powder,” which acted “against all Malignant, and Pestilent Diseases” (Woolley, 1675a, p.133). The Countess of Kent was Elizabeth Grey (1581-1651), author of the 1653 book *A choice manual, or, rare and select secrets in physick and chirurgery*. This recipe in turn referred to an unnamed “professor of medicine” (Grey, 1653, p.198), alerting us to the circulation of knowledge between licensed physicians and literate upper-class women. Besides the refined ingredients indicated such as pearls and coral, Woolley mentioned “Oriental Bezoar” and musk.^7^ Interestingly, the powder was meant for use by women and children, which again attests to the author’s view of the healing role of women.

The other mention was of “Gascoign Powder,” a panacea recommended against fever, measles, plague, and melancholy. This preparation was intended for a more restricted group, considering the expensive ingredients: not only oriental bezoar, but also crab eyes and pearls. Although Woolley cited these two powders as different recipes, it is important to note that Gascoigne’s powder was also known as the Countess of Kent’s powder, according to the historian Lucy Moore (2018, p.4).

Besides the panaceas that used spices, the text presents another pharmacological recipe that was well-known since ancient times: theriac. This preparation was mentioned in parts one and two, and referred to by two different terms: “treacle” and “Theriacal extraction.” Theriac dates back to Galen’s time, and this compound spread widely among Arab Muslims and Christians in the following centuries. It was taken orally or used as a salve, and was originally believed to be an antidote against venom; over time, it became a panacea and was administered until at least the nineteenth century (Prioreschi, 1998, p.441).

Venice was one of the places known for the production of theriac, and Woolley mentions its preparation in part two in the recipe for “Plague-water” (recipe 52). A similar recommendation appears in part one (recipe 98), also against plague. In this case, Woolley highlighted “Andramachus treacle,” named for the Emperor Nero’s court physician who created theriac, according to Galen (Vogt cited in Hankinson, 2008, p.312). This preparation was also known as Venice treacle or “galena” (Flanagan, 2001, p.12). In any case, we can assume that Woolley was referring to the same compound.

As we can see, several of these healing waters were accompanied by mentions of their creators. The physicians were not cited for no reason: linking medicines to their creators was a way of demonstrating “their long history of proven effectiveness” (Allen, 2016, p.101). Besides reinforcing the credibility of her recipes, Woolley employed a common practice in medicinal recipe books of the time by using the term “*probatum*” or “proved.” Although this was a medieval tradition more concerned with theoretical than practical appeal (Stobart, 2016, p.48) and did not necessarily attest to the author’s experiences, we must recognize the confidence such an annotation could create among readers.

Besides purgatives and panaceas, another widespread therapy in the theory of humors involved baths. For example, in part two, Woolley presented a recipe involving a bath followed by a tampon to cure hemorrhoids. The bath contained another foreign ingredient, *Cassia fistula*, and was directed at postpartum women. Besides this Asian plant, the bath also contained absinthe and wine, and was to be utilized after delivery. Following the bath, a tampon with aloe powder mixed with pennyroyal oil was to be utilized. Pessaries had been prescribed for women since at least Ancient Greece (Miles, 2004, p.82). Although Galen described more treatments for men, some therapies were specifically for women, such as those employing pessaries. Because of the intimate nature of their use, pessaries were commonly the responsibility of midwives, who may have been among Woolley’s readership.

Another frequent therapy in classical ancient medicine that likely originated in Egypt was the clyster or enema (Prioreschi, 1996, p.403), which consisted of intestinal cleansing by introducing liquid into the rectum and colon. This process was recommended in recipe four, for “Griping of the Guts.” Significantly, Woolley blended this recipe with culinary ingredients (like anise seed, fennel, pomegranate, and egg yolk) together with laudanum, a medication developed in Renaissance Europe.

Although her work was widely based on medical recommendations originating with Hippocrates and Galen, we can also find approaches involving medical innovations from early Modern times. These include chemical preparations such as laudanum (from the Latin *laudare,* to praise) proposed and used since the sixteenth century by Paracelsus (Philippus Aureolus Theophrastus Bombastus, c.1493-1541) (Porter, 2006, p.258). As noted above, this medication was mentioned in recipe four to address intestinal problems; laudanum generally consisted of a mixture of opium and alcohol, and was administered in a variety of circumstances (Miller, 2014, p.167). In Woolley’s recipe, it was to be dissolved into spirit of mint for subsequent use as a clyster (Woolley, 1675a, p.128).

Other chemical preparations recommended in the book utilized absinthe and turpentine. The plant from which absinthe is derived has been known since ancient times, particularly for its antiparasitic function; its English name, wormwood, refers to this property. The drink made from this plant, absinthe, spread across Europe during the Renaissance. Besides its recreational uses, advances in the process of distillation “purified and concentrated” the flavor of this drink, which further boosted sales alongside recommendations by physicians and pharmacists (Choffnes, 2016, p.116). Turpentine is extracted from coniferous trees, and was mentioned in four recipes. In the recipe to prevent miscarriage, Woolley (1675a, p.134) recommends “Venice Turpentine,” which mentions the city that produced this substance since medieval times and became “one of the principal markets for this medicinal drug” (Bilia et al., 2014, p.1).

Since many culinary and medicinal recipes circulated in handwritten form and often without indications of authorship, it is difficult to trace mentions of Woolley’s books. But another manuscript from the seventeenth century by Margaret Baker reveals similarities to the text analyzed here. The recipe for fennel oil in Baker’s text, for example, is almost identical to Woolley’s (Jeans, 2019, p.141). Since Woolley’s work was published and well-known by the late seventeenth century, it is possible that Baker copied this recipe.

## Women’s medical care

Woolley’s medicinal recipes also reveal an important topic in the medical practice of this period: the roles attributed to gender. In the case of women, care always revolves around two factors: how they were treated as patients, and their work as healers. These functions in turn were linked to social class. After all, women with financial means were able to exercise certain options of choice in this scenario, whether this meant consulting with learned physicians or learning medicinal recipes. These options were much more restricted for women in the lower classes.

Although women could be affected by various diseases, Woolley concerned herself with recommending resources for “female diseases,” namely those related to obstetric and gynecological conditions. Since women might be uncomfortable subjecting their bodies to examination by male hands (Code, 2000, p.233), the text offered ways for women to treat themselves. This is because, besides the taboos related to male examination of the female body, it is likely that specialized medical treatment was not the first option for many patients at that time. We consequently should consider that home treatment “was the only form of medicine available to most people in early modern England” (Wall, 2002, p.165).

Although Woolley did not utilize terms such as “practitioners” or “healers,” it is clear that the recipes were intended to be prepared by women and used by them or by their patients. A clear example is found at the start of the book, where Woolley (1675a, s.p.) states that ladies would find the text a guide “to help your Practice.” The recipes that focus on women’s bodies can be divided into three large groups: menstrual care, remedies related to conception, preventing miscarriage, and the postpartum period, and frequent diseases such as cancer. The author also added guidance on common childhood illnesses and conditions such as worms, fevers, and teething. Finally, there was a single recommendation for wetnurses in order to combat fever in infants (Woolley, 1675a, p.130). Because these women played an important part in infant care and health, they were carefully chosen, which is evident in manuals containing advice about wetnurses during this period (Harrison, 2016, p.93).

Because at that time fever was understood to be more of a disease than a symptom of an illness, Woolley presented specific advice about fever and even malaria in children. Again, many common ingredients in Hippocratic and Galenic medicine were present. In this case, the directives were to be followed by the wetnurse and the child. The wetnurse was to drink a frequent remedy: wine, with the addition of powdered crystal. Since wine was considered hot and wet, the same characteristics seen in children, these patients were not to consume this drink. Instead, the suggestion was topical: “Root of Devils-Bit” was to be hung around the child’s neck (Woolley, 1675a, p.130).

Along with self-care for women, it is clear that Woolley’s intent was to instruct women who needed to provide assistance to people in their families or communities. The word “patient” is used in 18 recipes, and “sick” in another two. In part one, “patient” is used in a recommendation for a cough syrup (Woolley, 1675a, p.153). In part two, the instructions include various preparations against everything from fever and dry cough to pleurisy and recent blindness in men (p.164). This book does not contain recommendation for women’s healing, but this does appear in another text. *The gentlewoman’s companion* mentions “an approved Medicine of London-Midwifes” to heal sore breasts (Woolley, 1673, p.169).

The sources of medical knowledge Woolley used (for empirical as well as erudite knowledge) can be seen, since physicians’ names as well as therapeutic recommendations are frequent in both texts, from 1675a and 1673. Furthermore, since she herself defended women’s right to education, female influence was also emphasized in her text, as in the case of the recipe for “the Countess of Kents Powder” (Woolley, 1675a, p.132). Finally and importantly, we must underscore her own learning in the healing arts. Her empirical training in this area is clear in the text of *The gentlewoman’s companion*. Although she recognizes the excellence of many authors in the subjects approached in this book – and includes their names in her recipes –, Woolley (1673, s.p.) states that this book was the “Product of my Thirty years Observations and Experience.”

In the supplement to *The queen-like closet*, we can better assess this process of medical learning and healing practice she cites. The author states that she acted as “Physician and Chirurgion” in her own home and to the benefit of her neighbors (Woolley, 1684, s.p.). She also reveals that this line of learning traced back to her mother and older sisters, who were “very well skilled in Physick and Chirurgery” and from whom she had learned some of these arts (p.8). The period during which she worked as a governess is also notable, since Woolley (p.8) took advantage of her ability to purchase the ingredients necessary to prepare “Balsoms, Salves, Oyntments, Waters for Wounds, Oyls, Cordials, and the like.” The lady in question also sought instruction from her physicians and surgeons in order to answer Woolley’s questions, and purchased books on the topic. These empirical activities linked to her study of medicine justified the cures she suggested and the title she utilized in this text: practitioner (p.8).

Her work included midwifery, a role common to women during this period. Woolley recalled when at the age of 22 she was called to attend a woman in labor. The patient was exhausted from the pain and “she fell into strong Convulsion fits” (Woolley, 1684, p.9) that threatened the life of both mother and baby. But thanks to the care received, the pain ceased and the delivery proceeded. After her first marriage to a teacher, Woolley practiced her healing arts on the children in her husband’s school, where she treated illnesses like fevers, malaria, and smallpox. And she added an observation: “unless [the boarders] were desperately ill” their parents trusted her work and she was able to heal them “without the help of any Physician or Chirurgion” (p.9).

The list of cases Woolley cites reveals two important points. Although most of her care was directed toward women, her therapeutic work also included men. Examples of the care addressed to male patients (whether lower-class or aristocratic) are cited in the supplement to *The queen-like closet* (Woolley, 1684, p.10). The second point relates to her surgical skills. Bodily intervention was the last therapy proposed in the theory of humors because of the damage it could cause, and was not widely recommended even by learned physicians. But the urgency of cases and her patients’ lack of resources motivated Woolley as she “acquired surgical skills” (Nagy, 1988, p.63) and sought to attend those who sought such care.

As mentioned earlier, the author consulted the knowledge of learned doctors and cited them by name throughout the 1675 text (*The accomplish’d lady’s delight...*). And in the supplement to *The queen-like closet*, physicians who were fundamental to the construction and practice of medicine popular at that time were also mentioned. Galen, Hippocrates, and Paracelsus were denoted “the Wisest Philosophers” (Woolley, 1684, s.p.). Even so, we must remember that Woolley was an empirical practitioner without academic training, and as such was part of a group long viewed with suspicion by licensed doctors.

Physicians who trained at university often viewed female practitioners as charlatans that caused more harm than good to their patients (Whaley, 2011, p.131). These healers included midwives. It is important to note that until the nineteenth century, midwives could be divided into two groups: urban and traditional. While the former group did have some degree of learning, the latter had “no formal training” (Whaley, 2011, p.92). In seventeenth-century England, licensing of midwives was the responsibility of the bishops, who only permitted those women who obtained a certificate from church chaplains to practice; this requirement had much more to do with character and orthodoxy than medical ability. Although we cannot precisely know how many midwives worked in England in the seventeenth century, we can assume that many, particularly in rural areas, did not have a “license.” These women were more vulnerable to potential accusations of using magical powers.

Women who had knowledge and experience in preparing medicinal plants were not only seen as ignorant by learned physicians but also subject to persecution by state and religious authorities. Although these wise women (and occasionally men) played an important role in traditional healing, efforts to remove these groups from medical practice began from the Renaissance period. As such, by suppressing “spiritual dimensions” in medicine and science, “healers, magicians, and witches lost their claim to manipulate the spiritual forces of the world” and “the ground was prepared for a mechanization of the world” (Ruggiero, 1993, p.17).

Although Woolley was not a licensed doctor, her daily practice seems to have occurred in a much more favorable setting than that of poor, illiterate healers who lived far from the big cities. In fact, there was a social recognition that linked domestic culinary tasks to the art of preparing remedies. In other words, a “good wife” was expected to make provisions to keep her family healthy. This network of knowledge involved her “mother, relatives, neighbours, medical practitioners,” and naturally, recipe books (Wear, 2000, p.52). In this sense, medicine that originated in literate doctors could be appropriated and transformed in domestic use, a practice that we can clearly see in Woolley’s books. This can help us to understand the absence of any statements by this author about accusations or persecution by lettered physicians or medical institutions.

## Final considerations

Even if Hannah Woolley (1675a, s.p.) recognized that many books on the topics of cooking and medicine were published at the time she was writing, her initiative was bold: to offer “all the Accomplishments necessary for Ladies.” Besides gathering beauty tips and advice, her text focused on women’s skills in healing themselves and others through written instructions. In order to not lose sight of the scenario where this production occurred, we must add that the culinary and medicinal recipe genre highlighted female authority, allowing women to express their identity in “a profoundly religious Society” that discouraged individual identity and “self-celebration” (Field cited in Down, Eckerle, 2007, p.59). The importance of female protagonism is notable in the dissemination of knowledge as well as in its practical implementation.

Despite these tools to construct this medical knowledge among women, we cannot minimize the turbulent arena in which empirical practitioners worked in seventeenth-century England. In 1684, the president of the College of Physicians, doctor Charles Goodall, wrote a book especially dedicated to fighting against “Empiricks and unlicensed practisers” (MacLennan, Pendry, 2011, p.6), which indicates that there were enough such people to provoke a response from medical institutions. Although Woolley combined various roles equivalent to physician, surgeon, pharmacist, and midwife, she did not write a single word about possible sanctions or persecution. Nor was the sale of remedies by Woolley the subject of dispute by learned physicians, characters who were interested in regulating this commercial activity and protecting it against lay practitioners (Marchitello, Tribble, 2017, p.212). As we have maintained in this article, it is possible that her social class and the medical principles of the time that she adopted also acted to impede any hostile behavior.

In this sense, we must be cautious with the notion that traditional female medical knowledge was essentially different from normative medicine, and that women practitioners were widely “suppressed by male physicians” (Ehrenreich, English cited in Weber, 2003, p.359); such positions are not supported by what we have read in Woolley’s texts. Quite to the contrary: the recipes and treatments she recommends are seen to be in line with the concepts of Hippocratic and Galenic medicine, besides revealing the influence of modern chemical medicine (as we have seen in some recipes). However, we must consider the impact that the theories disseminated in medical schools attained in popular medicine, and especially consider the possibility of an exchange of ideas and therapies between learned physicians and literate practitioners without academic training.

One caveat is important about employing medical theories with such distinct suppositions. Even though Paracelsus opposed ancient medicine and the principles of Galenic theory, the chemical aspect gained ground among physicians in England during the seventeenth century. So alongside the presence of the philosophical foundations of Galenic medicine, the emergence of other groups defending “experimental, chemical, mathematical and mechanical approaches to nature” can be noted (Wear, 2000, p.358). This helps us to understand a possible combining of Galenist and Parecelcist therapies by some physicians and empirical practitioners who did not necessarily see a contradiction between these theories.

Finally, we must recall the crucial role that women played in their communities and in society at that time. Woolley and other empirical practitioners occupied a space in which learned physicians were seldom present. Although some doctors did attend patients for reduced fees, documents of the time show “few examples of doctors who charged no fee to poor patients” (Nagy, 1988, p.30). Woolley and her colleagues treated those patients who could not pay for the services of a licensed doctor, or who were not content with the solutions such professionals proposed. Woolley made this clear in the supplement to *The queen-like closet,* where she stated that she cured the injured leg of a mason, since “because he was poor his Chirurgion gave it over” (Woolley, 1684, p.12). She also treated a “poor Woman” with a wound on her leg who had been by advised by the surgeon to amputate the limb (p.12).

These are just two examples of many reported by Woolley. She was yet another character in this important group of healers from different social classes and working in various areas of healing, from labor to postpartum, from creating cures from herbs to bedside treatment. All these women constantly “engaged in healing, assisted in childbirth, and comforted the dying” (Mack, 1992, p.29): activities vital to keeping people alive but at the same time daunting.

## NOTES

1 Briefly summarized, Galen’s “theory of humors” stated that bodily health resulted from balance between the four humors (phlegm, blood, yellow bile, and black bile). This balance depended on the patient’s age and diet, as well as the season of the year and the environment. The system that included the relationship between the four humors, the four elements (earth, air, water, and fire), and the four seasons could also include other factors such as “four flavors, four colors, and in late Antiquity, the four temperaments” and the astrological signs. See Nutton (2013, p.83).

2 Elaine Hobby casts doubt upon the authorship of this book, especially due to an autobiographic section of the text which more closely follows the conventions of the novel than the type of narrative of post-Restoration women writers. On this, see Hobby cited in MacLean (1995, p.179-200).

3 After completing this article, I learned of some work questioning the authorship of this book. Still, such questions do not invalidate this discussion of medicinal recipes and the role of women healers, since this article is also based on other publications from the period. Furthermore, the use of Woolley’s name in the book only confirms her popularity within the scenario of medicinal recipe publication.

4 For this and other quotes from non-English texts, a free translation has been provided.

5 On the presence of spices in medicinal recipes, see Polónia et al. (2016).

6 The date and place of birth for Charles Willoughby have not been established with certainty. Percy Kirkpatrick (1923) states he was accepted to the University of Padua in 1663-1664 to obtain the title of Doctor of Medicine and a year later returned to Oxford.

7 Bezoars are collections of undigested materials found in the digestive tract of mammals, reptiles, and even fish. According to Maria do Sameiro Barroso (2013), bezoars were introduced to western medicine by Arabic physicians around the twelfth century. Their curative powers, especially against fever, created a magical aura around these objects.
